# Effects of exercise training on brain metabolism and cognitive functioning in sleep apnea

**DOI:** 10.1038/s41598-022-13115-2

**Published:** 2022-06-08

**Authors:** Linda M. Ueno-Pardi, Fabio L. Souza-Duran, Larissa Matheus, Amanda G. Rodrigues, Eline R. F. Barbosa, Paulo J. Cunha, Camila G. Carneiro, Naomi A. Costa, Carla R. Ono, Carlos A. Buchpiguel, Carlos E. Negrão, Geraldo Lorenzi-Filho, Geraldo Busatto-Filho

**Affiliations:** 1 Escola de Artes, Ciencias e Humanidades, Universidade de Sao Paulo, Av. Arlindo Béttio, 1000 Ermelino Matarazzo, Sao Paulo, SP CEP: 03828-000 Brazil; 2 Departamento de Psiquiatria, Hospital das Clinicas HCFMUSP, Faculdade de Medicina, Universidade de Sao Paulo, Sao Paulo, SP Brazil; 3 Instituto do Coracao (InCor), Hospital das Clinicas HCFMUSP, Faculdade de Medicina, Universidade de Sao Paulo, Sao Paulo, SP Brazil; 4 Departamento de Radiologia e Oncologia, Hospital das Clinicas HCFMUSP, Faculdade de Medicina, Universidade de Sao Paulo, Sao Paulo, SP Brazil; 5 Escola de Educacao Fisica e Esportes, Universidade de Sao Paulo, Sao Paulo, SP Brazil

**Keywords:** Rehabilitation, Respiration

## Abstract

Impaired glucose metabolism reflects neuronal/synaptic dysfunction and cognitive function decline in patients with obstructive sleep apnea (OSA). The study investigated the extent to which exercise training (ET) improves cerebral metabolic glucose rate (CMRgl) and cognitive function in patients with OSA. Patients with moderate to severe OSA were randomly assigned to ET (3 times/week, n = 23) or no intervention (control, n = 24). Echocardiography and apolipoprotein ε4 (APOEε4) genotyping were obtained at baseline. Both groups underwent cardiopulmonary exercise testing, polysomnography, cognitive tests, brain magnetic resonance imaging, and ^18^F-fluoro-2-deoxy-d-Glucose positron emission tomography (^18^FDG-PET) at baseline and study end. Compared with control, exercise-trained group had improved exercise capacity, decreased apnea–hypopnea index (AHI), oxygen desaturation and arousal index; increased attention/executive functioning, increased CMRgl in the right frontal lobe (*P* < 0.05). After ET an inverse relationships occurred between CMRgl and obstructive AHI (r = − 0.43, *P* < 0.05) and apnea arousal index (r = − 0.53, *P* < 0.05), and between the changes in CMRgl and changes in mean O_2_ saturation during sleep and non-rapid eye movement sleep (r = − 0.43, *P* < 0.05), desaturation during arousal (r = − 0.44, *P* < 0.05), and time to attention function testing (r = − 0.46, *P* < 0.05). ET improves OSA severity and CMRg in the frontal lobe, which helps explain the improvement in attention/executive functioning. Our study provides promising data that reinforce the growing idea that ET may be a valuable tool to prevent hypoxia associated with decreased brain metabolism and cognitive functioning in patients with moderate to severe OSA.

**Trial registration:** NCT02289625 (13/11/2014).

## Introduction

Obstructive sleep apnea (OSA) is characterized by complete or partial obstruction of the upper airways causing sleep fragmentation and intermittent hypoxemia and is associated with several adverse cardiovascular consequences^1–3^. There is growing evidence that untreated OSA may also contribute to cognitive dysfunction, including attention/executive functioning by mechanisms that are not completely understood.

OSA has been associated with reduced regional cerebral metabolic rate of glucose consumption (CMRgl) measured by ^18^F-fluoro-2-deoxy-d-Glucose positron emission tomography (^18^FDG-PET) particularly in the frontal lobe. CMRgl is an early marker of neuronal injury and synaptic dysfunction^4^ and a reliable predictor of cognitive decline^5^. In addition, one observational study found reduced CMRgl in the precentral gyrus and the cingulate cortex in patients with OSA that modestly improved after treatment with continuous positive airway pressure (CPAP)^6^. These clinical observations are confirmed by the experimental study that showed a decrease in dendritic branching and alterations in dopaminergic pathways in the frontal lobe in a rodent model of intermittent hypoxia^7^. Frontal lobe dysfunction helps to explain cognitive deficits, such as inattention, learning difficulties, and poor executive functioning in patients with OSA^8,9^. Results from other studies^10–12^ have also found evidence of poorer cognitive performance in OSA patients with genotyping of the apolipoprotein ε4 polymorphic allele (APOE ε4) than non-carriers with OSA. The higher apnea–hypopnea index (AHI) was associated with worse executive functions and language skills among APOE ε4 carriers^12^. In the previous study, including patients with an AHI ≥ 15 events per hour of sleep, the genotype frequency was 23.8% for the ε4 genotype^13^.

Exercise training (ET) is an attractive treatment for the typical sedentary patient with OSA because ET decreases OSA severity and improves sleep quality^14,15^. In addition, ET has pleiotropic beneficial effects and improves several cardiovascular pathways known to be affected by OSA. Among patients with OSA, ET reduces sympathetic overactivity and improves vascular function at rest and during exercise^16^ or mental stress^17,18^, and cardiac autonomic modulation^19^. ET may also afford brain protection in patients with OSA^20^. However, few studies have controlled the APOE ε4 allele which can significantly affect the brain function^21,22^ and is known to be associated with worse cognitive functioning^23^. The aim of this present randomized study was to investigate the extent to which ET improves cognitive function and CMRgl in patients with OSA. We test the hypothesis that ET improves cognitive functioning and CMRgl in recently diagnosed sedentary patients with OSA, taking into consideration APOE ε4 genotyping.

## Methods

### Participant’s eligibility and trial overview

Male and female individuals, 40 to 65 y of age, were recruited from the community enriched by relatives and friends from the staff of the Heart Institute Hospital. This is the primary report of the study designed to evaluate brain metabolism and cognitive function. Some substudies using a sub set of patients of the present cohort evaluating ET on muscle metaboreflex control^16^, cardiac autonomic modulation^19^ and sympathetic nerve activity^17^ have been previously reported. Subjects who had body mass index (BMI) > 40 kg/m^2^, cardiopulmonary disease, chronic renal disease, diabetes mellitus, atrial fibrillation, pacemaker, renal failure, echocardiographic evidence of impaired left ventricular function (ejection fraction < 45%), history of severe psychiatric disorders, dementia or other neurodegenerative disorders, smoking or alcohol abuse (two or more drinks per day), any sleep apnea treatment, sleep disorders other than OSA, circadian desynchrony (eg, shift workers), less than 2 years of formal education, use of psychoactive substances, auditory or visual disorders, claustrophobia were excluded from the study. All subjects were sedentary adults who had not exercised regularly for at least 3 months before enrolling in this study. This protocol was approved by Scientific Committee of the Heart Institute (Instituto do Coração), Hospital das Clínicas da Faculdade de Medicina da Universidade de São Paulo, and by the Ethics Committee for Research with Human Beings (0833/10) of the Clinical Hospital, University of São Paulo Medical School, and all subjects gave written informed consent. All methods were performed in accordance with relevant guidelines and regulations.

All subjects underwent evaluation of biochemical blood profile, echocardiography, and APOE ε4 genotyping at baseline. Genotypes for APOE ε2/ε3/ε4 were determined by polymerase chain reaction followed by restriction fragment length polymorphism analysis^24^. Full nocturnal polysomnography, maximal exercise capacity, neuropsychological evaluation, brain magnetic resonance (MR) imaging and FDG-PET were performed at baseline and at the end of the study.

### Exercise training protocol

The ET program consisted of three 60-min, supervised, exercise sessions per week. Each session consisted of 5 min of stretching, 25 min of cycling on the ergometer bicycle in the first month and up to 40 min in the last 5 months, 10 min of local strengthening exercise, and 5 min of cool down with stretching exercises. The cycling exercise intensity was established by heart rate levels that corresponded to an anaerobic threshold up to the respiratory compensation point obtained in the cardiopulmonary exercise test. During the exercise sessions, when a training effect was observed, as indicated by a decrease of 8% to 10% in heart rate, the bicycle workload was increased by 0.25 or 0.5 kpm throughout the training until reaching the target heart rate^16^. Using this same strategy of exercise program (with both aerobic and strength components), we previously demonstrated several benefits in neurovascular and sleep parameters in patients with heart failure and sleep apnea^25^ and in patients with obstructive sleep apnea without other comorbidities^16^. Exercise compliance was assessed as percentage of exercise sessions attended.

### Outcome measures

Age, sex, educational level, compliance, genotyping of the APOE ε4 were all registered. Medication was also recorded for each patient. The echocardiographic study was performed using a Vivid E9 machine (GE Healthcare, Wauwatosa, WI). The left ventricular ejection fraction were calculated using Teichholz’s method. Clinical blood pressure (BP) readings were obtained from the left arm of subjects while seated, after 5 min of quiet rest, with a mercury sphygmomanometer. The subjects were classified as normotensive if the average systolic and diastolic BP levels were < 140 or 90 mmHg. Resting heart rate was evaluated by electrocardiography. All participants underwent overnight polysomnography (Embla N7000, Medcare Flaga, Reykjavik, Iceland) as previously described^26^. The sleep stages and respiratory events were scored according to the criteria of Iber et al.^27^. Apnea was defined as a ≥ 90% drop in respiratory amplitude, lasting at least 10 s. Hypopnea was defined as at least 50% drop in respiratory amplitude, lasting at least 10 s, associated with O_2_ saturation declines ≥ 3% or arousals (acceptable criteria). The AHI was defined as an index of the number of apnea and hypopnea events per hour of sleep. All sleep parameter analysis was identified by visual inspection conducted by a single investigator, blinded to the treatment allocation. Maximal exercise capacity was determined by means of a maximal progressive cardiopulmonary exercise test (Vmax Analyzes Assembly, Encore 29 System, VIASYS Healthcare Inc, Yorba Linda, CA, USA) on an electromagnetically braked cycle ergometer (Via Sprint 150P, Ergoline, Bitz, Germany), as previously described^16^.

#### Primary outcome

The primary outcome of the study was to measure the effects of ET on regional CMRgl measured by 18F-fluoro-2-deoxy-d-Glucose positron emission tomography (18FDG-PET). All participants underwent 18FDG-PET, within 1 week after the brain MR imaging acquisition was performed. PET data were acquired by using a dedicated lutetium oxyorthosilicate-16-section FDG-PET scanner (Biograph-16; Siemens, Erlangen, Germany). Following exactly the same protocol before and after follow-up, we determined blood glucose levels after at least 12 h of fasting; then we administered an intravenous injection of 370 MBq (10 mCi) of FDG. Subjects remained in a quiet dimly lit room with their eyes closed, and 18FDG-PET imaging was initiated 60 min after FDG administration, by using the 1-bed-position 3D protocol with 15 min of acquisition. For neuroimage processing, 18FDG-PET images were co-registered to the T1-MR datasets of the same individual using the PMOD software tool (version 3.4, PMOD Technologies Ltd., Zurich, Switzerland). Coregistered 18FDG-PET images were corrected for partial volume effects (PVEs). All the T1-MRI and 18FDG-PET images were processing using Statistical Parametric Mapping, version 12 (SPM12; https://www.fil.ion.ucl.ac.uk/spm/), implement in MATLAB software (The Math-Works, Natick, MA, USA) (see Supplemental Digital Content 1. Text that includes more details about neuroimaging data acquisition and processing).

#### Secondary outcomes

The general screening measures of cognition using Mini-Mental State Examination (MMSE)^28^ was administered. Episodic verbal memory and learning abilities were assessed using Rey Auditory Verbal Learning Test (RAVLT5—sum of 5 recall trials of 15 words and RAVLT late − delayed recall after 30 min)^29^. Frontal assessment was evaluated using Frontal Assessment Battery^30^. Attention was evaluated using Trail Making Test—Part A^31^, Forward Digits^32^, Digit Symbol test^12^, Stroop Color Word Test (SCWT—Part 1 and 2)^33^. Inhibitory control was evaluated using SCWT—Part 3^33^. Updating was evaluated using Backward Digits^32^. Shifting or cognitive flexibility was evaluated using Trail Making Test—Part B^31^. All cognitive evaluation and data analysis was conducted by a single investigator, blinded to the study protocol (see Supplemental Digital Content 1. Text that includes more details about cognitive tests). Body composition was measured by bioelectrical impedance (Quantum II; RJL System Clinton Twp, MI). Intelligence Quotient (IQ) was estimated by using the Wechsler Abbreviated Scale for Intelligence (WASI)^34^. Participants also answered at baseline the Self-Reporting Questionnaire (SRQ-20)^35^, which investigates symptoms associated with common mental disorders, the Beck Anxiety Inventory^36^, and the Beck Depression Inventory^37^.

### Sample size and statistical analysis

For the sample size calculation of this study, the OpenEpi interface developed for epidemiological statistics^38^ was used. A power of 80% and a confidence interval of 95% were adopted, with an estimated value for a type I error of 5%. As there was no previous study investigating the effect of exercise on regional CMRgl, the means and standard deviation were considered based on our previous study investigating the effects of ET on sleep parameters in patients with sleep apnea^25^. As a final result, a value of 10 individuals in each group was obtained. However, because this was a new parameter, we target to recruit 25 patients in the exercise-trained and 25 in the control group. Sociodemographic information, clinical data, and CMRgl peak values were analyzed using STATISTICA 12 software (StatSoft Inc., Tulsa, OK). A χ^2^ (sex, medicine, differences in genotype frequencies), Mann–Whitney, unpaired or paired Student *t* tests were used to compare differences between groups at baseline or delta changes (follow-up—baseline). The 95% confidence interval (95% CI) was calculated using mean changes ± 1.96 * SE. Two-way analysis of variance with repeated measures (physical, hemodynamic and sleep parameters, cognitive functioning, functional capacity) was used to compare within and between group differences at baseline and after follow-up. In the case of significance, post hoc comparisons were performed using the Duncan multiple range test. A *P* < 0.05 was considered statistically significant. In the analyses, APOE ε4 variable, and time interval of PET measurements between groups were entered as covariates, as they may affect mainly brain metabolic measures and cognitive functioning. Pearson correlation analysis was used to examine the association between CMRgl with sleep parameters and cognitive measures (*see* Supplemental Digital Content 1. Text that includes more details about neuroimage statistical analysis).

## Results

**Figure 1:**
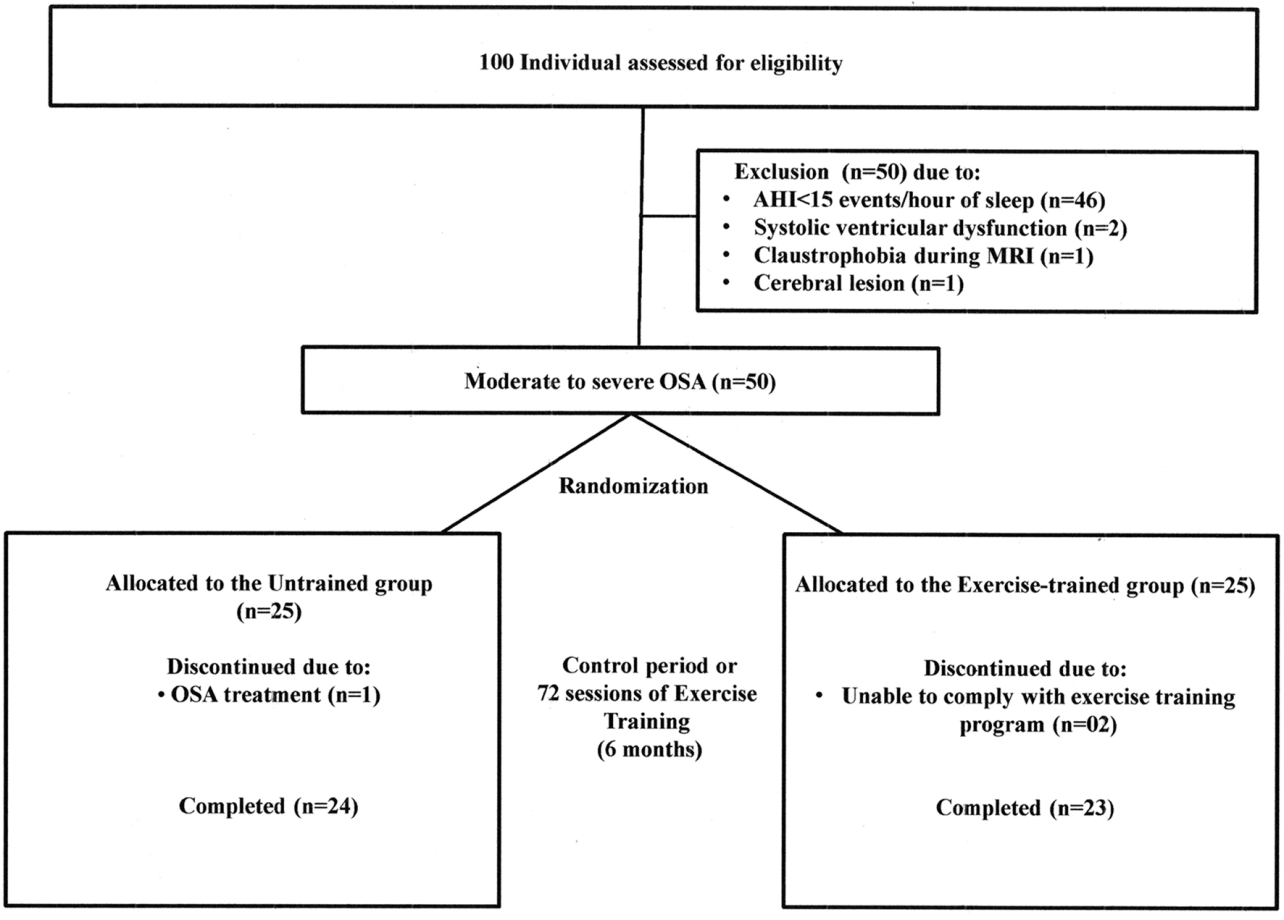
Profile of a randomized clinical trial showing the progress of patients throughout the trial. *AHI*, apnea–hypopnea index; *MRI*, magnetic resonance image; *OSA*, obstructive sleep apnea.

From a total of 100 subjects potentially eligible initially selected to participate in the study, 50 subjects selected were excluded due to no moderate to severe OSA (n = 46); 2 patients due to the presence of asymptomatic systolic ventricular dysfunction; 1 patient had claustrophobia during MR data collection; and 1 patient had a silent lacunar infarct detected by MR imaging. Fifty OSA patients were randomly assigned to either control (n = 25) or exercise-trained group (n = 25). Figure 1 presents the CONSORT flow diagram, including the progress of patients throughout the trial. Patients were randomly assigned to either a training group or a sedentary control group. Subjects were randomized on a one to one basis. That is, an individual was selected for the control group and next to the exercise-trained group and so on. After baseline measurements, patients the control group were frequently contacted by telephone and were instructed to avoid any regular exercise program. After the end of the protocol, the control group was invited to participate in the ET program or received guidelines for starting an ET program.

During protocol, one patient in the control group started treatment of OSA with CPAP and 2 patients failed to keep at least one ET session per week. Therefore, the final sample consisted of 47 subjects. Table 1 shows the baseline characteristics of the control and the exercise-trained OSA groups. The presence of at least one APOE ε4 allele was found in 7 subjects in the control group and 4 subjects in the exercise-trained group (*P* > 0.05). No significant baseline differences existed between groups in physical, metabolic, cardiovascular, sleep parameters, number of hypertensive patients, IQ, and medications.

**Table 1:** Baseline characteristics of the population studied.

	Control (n = 24)	Exercise (n = 23)	*P* value
Male, n	17	10	0.60
Age, y	51 ± 6	53 ± 7	0.29
BMI, kg/m^2^	29.3 ± 3.1	30.2 ± 3.8	0.36
Body fat, %	25 ± 8	30 ± 7	0.09
Education, y	12(5–20)	11(5–18)	0.81
IQ	85 ± 12	89 ± 13	0.22
SQR-20	5 ± 3	5 ± 4	0.73
BAI	6 ± 8	8 ± 7	0.38
BDI	7 ± 5	8 ± 5	0.43
Hypertension, n	3	3	
**Metabolic**			
Glucose, mg/dL	100 ± 10	105 ± 9	0.11
Total cholesterol, mg/dL	201 ± 42	204 ± 37	0.13
Peak VO_2_, ml/kg/min	25 ± 6	24 ± 6	0.33
**Cardiovascular parameters**			
Heart rate, beats/min	66 ± 9	68 ± 8	0.30
Systolic BP, mmHg	124 ± 13	123 ± 14	0.81
Diastolic BP, mmHg	79 ± 9	80 ± 7	0.69
LVEF, %	66 ± 3	68 ± 5	0.18
**Sleep parameters**			
Arousal index, events/h	31 ± 15	33 ± 17	0.44
AHI*,* events/h	41 ± 24	45 ± 29	0.46
O_2_ desaturation, events/h	32 ± 23	39 ± 29	0.23
**Apolipoprotein genotyping**			
Polymorphic allele ε4, n	7	4	0.12

Data are means ± SD.

*BMI* body mass index; *IQ* estimated intelligence quotient; *SQR-20* Self-Reporting Questionnaire; *BAI* Beck Anxiety Inventory score; *BDI* Beck Depression Inventory score; *VO*_*2*_ oxygen uptake; *LVEF* left ventricle ejection fraction; *BP* blood pressure; *AHI* apnea–hypopnea index; *O*_*2*_ oxygen.

### Effects of exercise training on physical and sleep parameters

Compliance with the exercise program (72 sessions or 100% of training) ranged from 85 to 100% of the exercise sessions attended for individuals with OSA. Physical and physiologic parameters were unchanged after follow-up in the control group (*P* > 0.05 for all parameters, Table 2). ET did not change BMI. The exercise-trained group had a significant decrease in percentage of body fat compared with the control group (*P* < 0.05). In both groups, resting heart rate and arterial BP did not change (*P* > 0.05). ET significantly increased peak oxygen consumption (peak VO_2_) (*P* < 0.05). No significant changes in peak VO_2_ were found in the control group. The comparisons between groups also showed that the delta changes (baseline – follow-up) in peak VO_2_ in the exercise-trained group were significantly greater than those observed in the control group (*P* < 0.05). No changes occurred in total sleep time and sleep efficiency between groups after intervention or control period. In contrast, arousal index decreased significantly in the exercise-trained group (*P* < 0.05). The comparisons between groups also showed that the delta changes in AHI, arousal index, and O_2_ desaturation in the exercise-trained group were significantly greater than those observed in the control group (*P* < 0.05).

**Table 2:** Effects of exercise training on physical characteristics, physical capacity, and sleep parameters in individuals with obstructive sleep apnea.

	Baseline	Follow-up	Changes	95%CI
**Physical characteristics**				
BMI, kg/m^2^				
Control	29 ± 3	29 ± 3	0.14	[− 0.27; 0.56]
Exercise	30 ± 4	30 ± 4	− 0.44	[− 1.0; 0.12]
Body fat, %				
Control	25 ± 8	24 ± 7	− 1.04	[− 2.78; 0.70]
Exercise	30 ± 7	27 ± 9*	− 2.65	[− 4.36; − 0.94]
**Metabolic parameters**				
Glucose, mg/dL				
Control	100 ± 10	103 ± 10	3.29	[− 1.64; 8.22]
Exercise	105 ± 9	110 ± 14	5.48	[1.94; 9.02]
Total cholesterol, mg/dL				
Control	201 ± 42	200 ± 44	− 1.6	[− 10.82; 7.57]
Exercise	204 ± 37	211 ± 41	7.3	[− 4.48; 19.09]
**Physiological parameters**				
Heart rate				
Control	67 ± 9	67 ± 9	− 0.43	[− 4.08; 3.21]
Exercise	68 ± 8	66 ± 7	− 2.52	[− 4.59; − 0.46]
Systolic BP, mmHg				
Control	123 ± 13	125 ± 15	− 1.75	[− 6.96; 3.46]
Exercise	120 ± 14	116 ± 13	2.61	[-1.77; 6.99]
Diastolic BP, mmHg				
Control	78 ± 9	78 ± 8	− 0.38	[− 5.03; 4.28]
Exercise	79 ± 8	78 ± 6	0.48	[− 2.38; 3.34]
VO_2_ peak, mL kg^−1^ min^−1^				
Control	25 ± 6	24 ± 6	− 2.60	[− 5.06; − 0.14]
Exercise	24 ± 6	28 ± 7*^†^	4.07^††^	[2.82; 5.31]
**Sleep parameters**				
Total sleep time, min				
Control	385 ± 49	391 ± 48	3.01	[− 14.41; 20.43]
Exercise	360 ± 59	370 ± 53	11.25	[− 13.87; 36.37]
Sleep efficiency, %				
Control	85 ± 7	85 ± 8	0.98	[− 2.53; 4.48]
Exercise	82 ± 10	85 ± 8	0.61	[− 7.53; 6.30]
AHI, events/h				
Control	41 ± 24	46 ± 29	3.02	[− 3.36; 9.40]
Exercise	45 ± 29	39 ± 24	− 5.36^††^	[− 10.48; − 0.44]
Arousal index, events/h				
Control	31 ± 15	31 ± 17	− 1.70	[− 7.09; 3.60]
Exercise	33 ± 17	26 ± 13^†^	− 7.50^††^	[− 12.74; 2.25]
O_2_ desaturation, events/h				
Control	32 ± 23	41 ± 27	8.45	[1.16; 15.74]
Exercise	39 ± 29	36 ± 24	− 3.47^††^	[− 8.81; 1.86]

Data are means ± SD and means of delta changes [95% CI].

*BMI* body mass index, *BP* blood pressure; *VO*_*2*_ oxygen uptake; *AHI* apnea–hypopnea index.

*P* value indicates comparisons between groups (2-way ANOVA).

*Within group comparison, *P* < 0.05.

^†^Between group comparisons in post intervention, *P* < 0.05 (detected by post hoc analysis).

^†^^†^Change between groups (Unpaired t test).

### Effects of exercise training on cognitive performance

**Figure 2:**
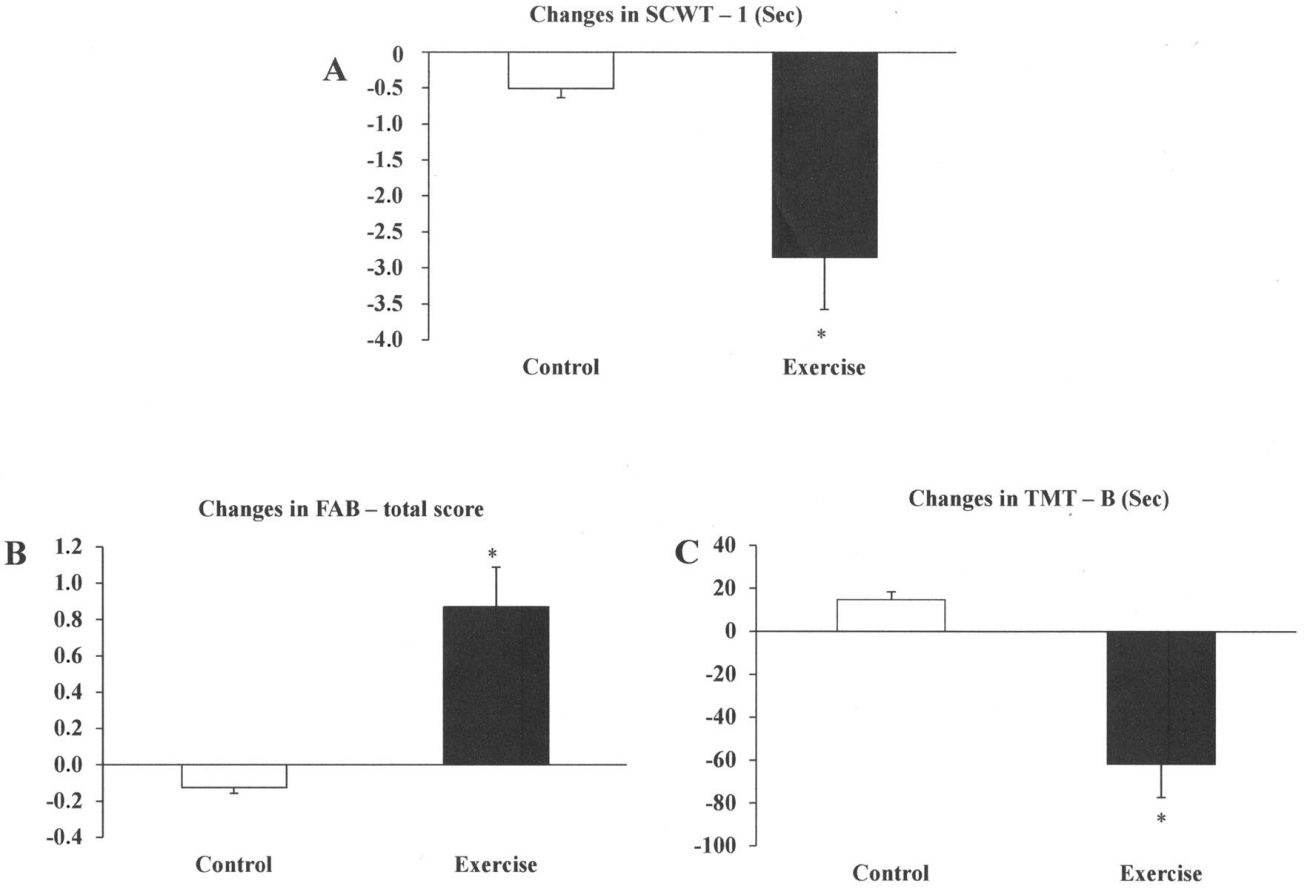
(**A**–**C**) Changes in cognitive performance in patients with obstructive sleep apnea in the control group and exercise-trained group. *CSWT*, Stroop Color Word Test; *FAB*, Frontal Assessment Battery; *TMT*, Trail Making Test. *P < 0.05 control versus training group.

There were no significant differences between the groups in MMSE and RAVLT (Table 3). In regard to attention and inhibitory control, significant differences between groups were observed in the SCWT—Part 1 and 3 at baseline and after ET. The SCWT—Part 1 decreased after intervention, only in the exercise-trained group. The changes in SCWT—Part 1 were significantly higher in the exercise-trained compared with control group (Fig. 2A).

**Table 3:** Attention, memory, and executive functioning among individuals with obstructive sleep apnea in the control and training groups at baseline and after follow- up, taking into consideration the APOE ε4 allele.

	Baseline	Follow-up	Changes	95%CI
**Global cognition**				
MMSE score				
Control	27 ± 3	27 ± 2	0.1	[− 0.76; 0.76]
Exercise	27 ± 2	27 ± 2	0.65	[− 0.28; 1.58]
**Frontal assessment**				
FAB-total score				
Control	16 ± 2.0	16 ± 1.7	0.13	[− 0.62; 0.37]
Exercise	16 ± 2.0	18 ± 1.8*^†^	0.87^††^	[0.16; 1.58]
**Episodic memory and learning**				
RAVLT A1-A5				
Control	41 ± 8	40 ± 7	− 0.17	[− 3.19; 2.86]
Exercise	40 ± 10	43 ± 9	1.13	[− 2.31; 4.57]
RAVLT late				
Control	8 ± 3	8 ± 3	− 0.08	[− 1.0; 0.83]
Exercise	9 ± 3	9 ± 3	0.04	[− 0.98; 1.07]
**Attention**				
TMT-A, sec				
Control	61 ± 47	64 ± 68	2.85	[− 12.86; 18.55]
Exercise	48 ± 27	42 ± 22	− 6.04	[− 14.01; 1.94]
Forward digits				
Control	6 ± 3	5 ± 3	− 0.17	[− 1.03; 0.70]
Exercise	5 ± 2	6 ± 2	0.35	[− 0.47; 1.16]
Digit symbol				
Control	32 ± 13	35 ± 13	3.63	[1.15; 6.10]
Exercise	38 ± 13	39 ± 14	1.35	[− 0.52; 3.22]
SCWT-1, sec				
Control	24 ± 22	23 ± 19	− 0.51	[− 2.12; 1.11]
Exercise	19 ± 5^#^	17 ± 4*^†^	− 2.86^††^	[− 4.36; − 1.36]
SCWT-2, sec				
Control	31 ± 18	28 ± 10	− 3.09	[− 7.51; 1.33]
Exercise	23 ± 5	21 ± 5	− 1.60	[− 2.81; 0.40]
**Inhibitory control**				
SCWT-3, sec				
Control	44 ± 25	40 ± 17	− 4.06	[− 12.96; 4.86]
Exercise	34 ± 8^#^	27 ± 7^†^	− 7.07	[− 8.87; − 5.28]
**Updating**				
Backward digits				
Control	4 ± 2	4 ± 2	− 0.17	[− 0.62; 0.29]
Exercise	4 ± 1	5 ± 2	0.43	[− 0.14; 1.01]
**Shifting**				
TMT-B, sec				
Control	176 ± 149	191 ± 160	14.79	[− 22.81; 52.39]
Exercise	195 ± 158	133 ± 68*^†^	− 61.78^††^	[− 107; 17]

Data are means ± SD and means of delta changes [95% CI].

*MMSE* Mini-Mental State Examination; *FAB* Frontal Assessment Battery; *RAVLT A1-A5* Rey Auditory Verbal Learning Test (sum of 5 recall trials of 15 words); *RAVLT late* delayed recall after 30 min; *TMT* Trail Making Test; *SWCT* Stroop Color Word Test.

*P* value indicates comparisons between groups (2-way ANOVA).

*Within group comparison, *P* < 0.05.

^†^Between group comparisons in post intervention, *P* < 0.05.

^#^Between group comparisons in pre intervention, *P* < 0.05 (detected by post hoc analysis).

^†^^†^Change between groups (Unpaired t test)**.**

Performance in the Frontal Assessment Battery and Trail Making Test—B significantly improved after ET. The delta changes in Frontal Assessment Battery (Fig. 2B), and Trail Making Test—Part B (Fig. 2C) were significantly higher in the exercise-trained group compared with controls. Forward and Backward Digits did not differ between groups before and after intervention.

### Effects of exercise training on cerebral glucose metabolism

**Figure 3:**
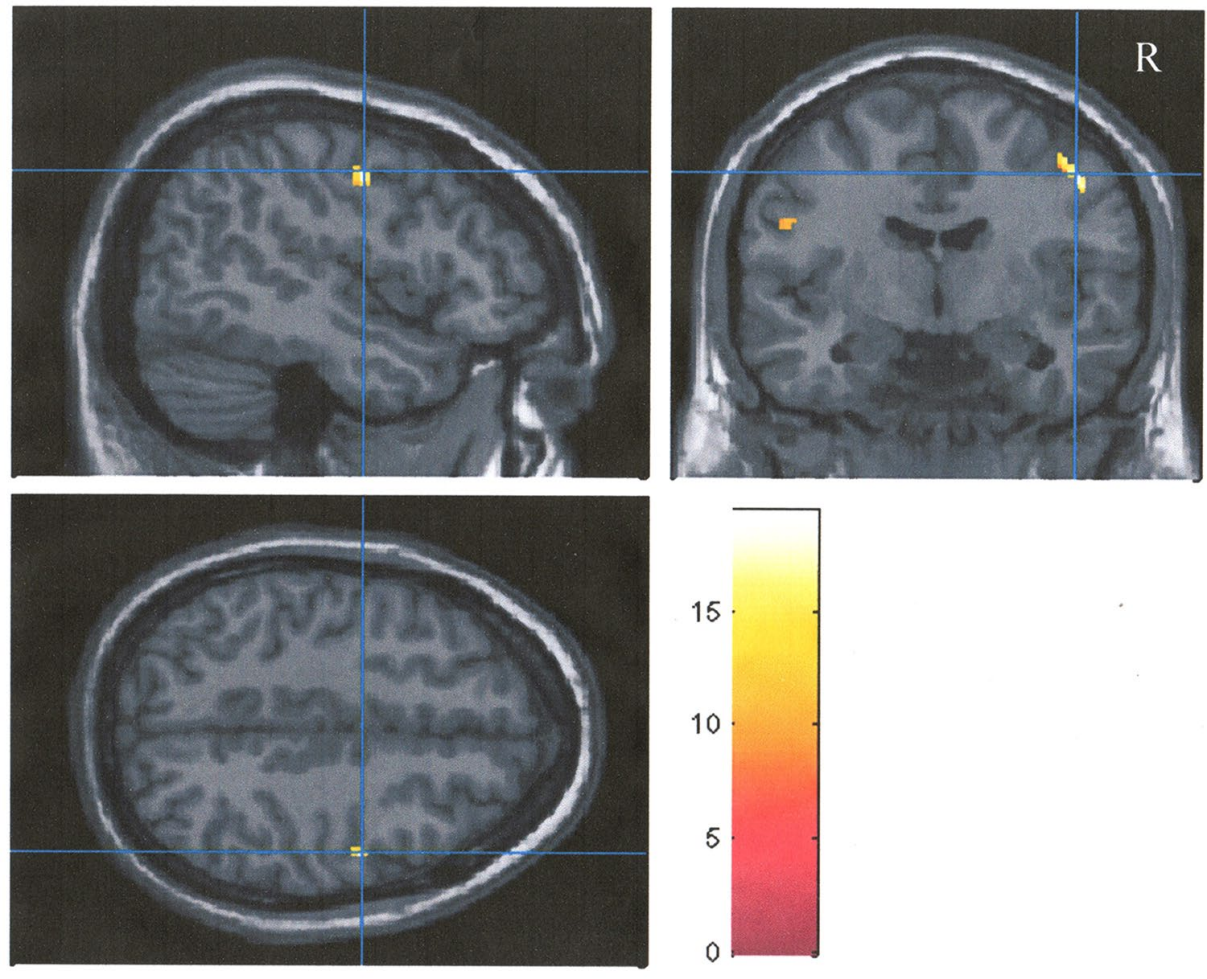
Findings showing clusters of changes in cerebral metabolic glucose rate (CMRgl) in the frontal lobe of the right hemisphere highlighted in yellow in exercise-trained and control patients with obstructive sleep apnea. Foci of significance are overlaid on sagittal, coronal, and axial brain slices spatially normalized in MNI space. All voxel clusters shown in the figures retained statistical significance after family-wise error correction for multiple comparisons (*P* < 0.005), corrected for multiple comparison over right frontal lobe and had a minimum extent threshold of 20 voxels. Statistical details are given in Table 4. The colored bar represents F-values. The model includes APOE ε4 allele and time interval between magnetic resonance data collection as covariates. R = right.

**Figure 4:**
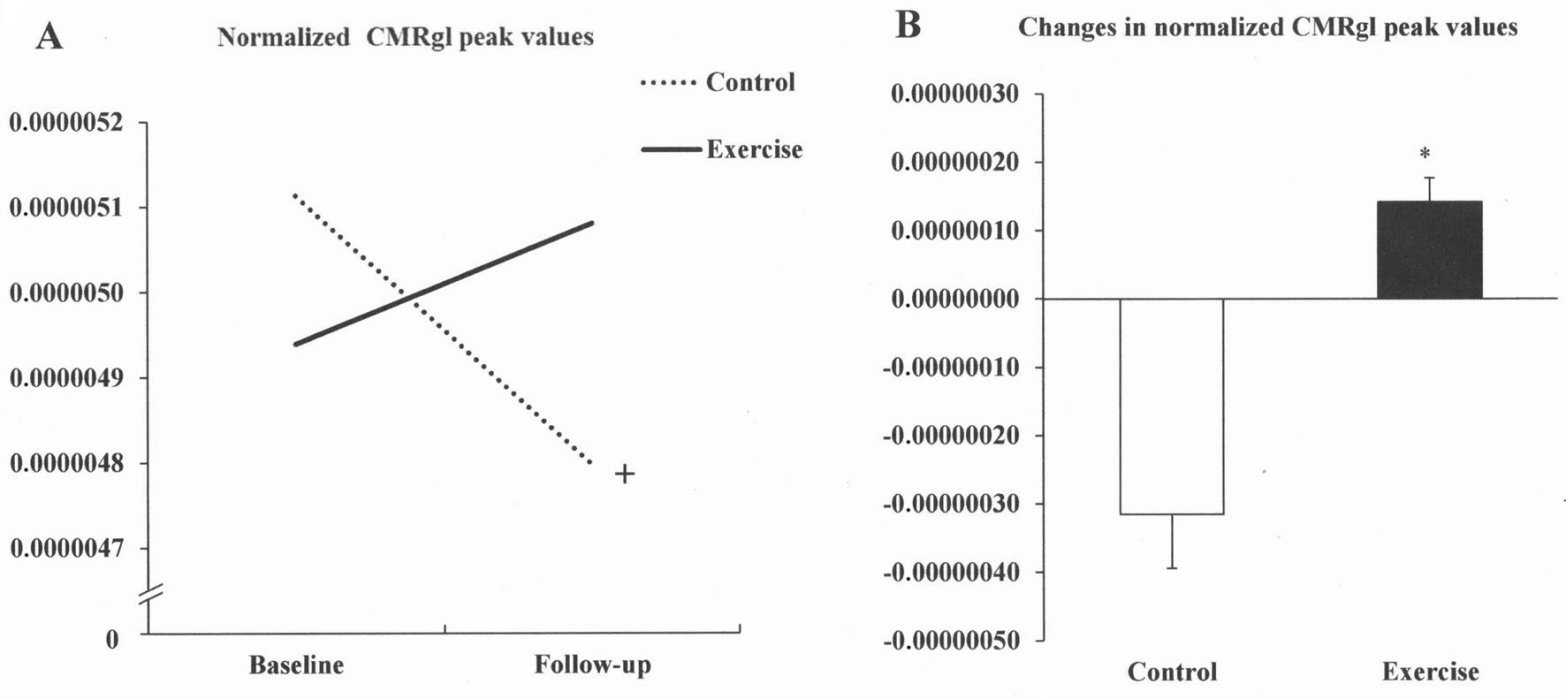
(**A**) Normalized cerebral metabolic glucose rate (CMRgl) peak values (voxel values extracted from the coordinate of maximal significance) within and between control and exercise-trained groups; (**B**) Delta changes in CMRgl peak values in patients with obstructive sleep apnea in the control group and exercise-trained group. ^+^*P* < 0.05 Significant difference compared with respective baseline values (2-way ANOVA). *P < 0.05 Control versus training group (Unpaired *t* test).

Using the hypothesis-driven with small volume correction approach, the voxel-wise comparison between groups revealed significant ANCOVA group x time interactions in the right frontal lobe (*P-corrected* < 0.05), (Table 4). Inspection of CMRgl peak values (voxel values extracted from the coordinate of maximal significance) in these clusters revealed that the exercise-trained group showed significant hypermetabolism in the right frontal lobe (*P-corrected* < 0.05) (Fig. 3). Absolute values (Fig. 4A) of CMRgl peak values showed that the control group had a significant decrease in CMRgl peak values. Further analysis using delta analysis (Fig. 4B) showed that peak brain glucose metabolism improved significantly in the exercise-trained group compared with the control group. These results were taking into account the confounding influence of patients with the presence of APOE ε4 allele. The mean interval weeks between PET measurements were different in the exercise-trained group compared with the control group. The time lag between neuroimage measurements was 27 ± 13 weeks in the control vs 40 ± 3.0 weeks in the exercise-trained group. This potential limitation was explored by the evaluation of volumetric and CMRgl differences taking also into account the interval (days between the first and follow-up MR data collection) as covariate in the ANCOVA model.

**Table 4:** Voxel-wise: longitudinal analysis taking into consideration the influence of polymorphic allele e4 of the apolipoprotein e and time interval in patients with obstructive sleep apnea undergoing exercise training or clinical follow-up.

Group comparison	Brain structure^a^	MNI coordinates^b^	Cluster size^c^	Peak Z score^d^	*P*FWE corrected^e^
Control vs. exercise	Frontal lobe (right)	48/− 6/46	45	3.80	0.046*

*FWE*, indicates family-wise error.

^a^Each region was circumscribed using the small volume correction approach, with anatomically defined volume-of-interest masks.

^b^MNI (Montreal Neurological Institute) coordinates of the voxel of maximal statistical significance with each cluster.

^c^Number of contiguous voxels that surpassed the initial threshold of *P* < 0.005 (uncorrected) in the statistical parametric maps.

^d^Z scores for the voxel of maximal statistical significance.

^e^Statistical significance after correction for multiple comparisons (voxel level).

*Refers to statistically significant differences.

The Pearson correlation coefficient between CMRgl with sleep parameters and cognitive functions did reach statistical significance only in the exercise-trained group. Inspection of regional metabolic peak values (voxel values extracted from the coordinate of maximal significance, corrected for the whole global mean value) revealed that significant inverse relationships existed between CMRgl and obstructive AHI (r = − 0.43, *P* < 0.05), apnea arousal index (r = − 0.53, *P* < 0.05), and changes between CMRgl and changes in mean O_2_ saturation during sleep and non-rapid eye movement sleep (r = − 0.43, *P* < 0.05), desaturation during arousal (r = − 0.44, *P* < 0.05), and SCWT—Part 1 (r = − 0.46, *P* < 0.05). No correlation coefficient was found between CMRgl with these parameters with the whole group or with the control group (*P* > 0.05).

## Discussion

In this randomized trial, we evaluated the effects of ET in recently diagnosed sedentary patients with moderate to severe OSA. Our study confirmed that ET improves functional capacity, OSA severity^39,40^, and attention/executive functioning. New important findings emerged from the study. First, ET promoted significant improvement in CMRgl in the right frontal lobe. Second, CMRgl was inversely associated with OSA severity and attention/executive functioning after ET.

ET increased CMRgl in the frontal area of interest that is related to attention/executive functioning, and is known to be the area strongly affected by intermittent hypoxia^41^. The increase in CMRgl is suggestive of improvement in synaptic activity, resulting from changes in functional and structural cellular mechanisms, which induce an increase of neuronal energy demand and in turn of glucose metabolism^42^. CMRgl depends on several factors including cerebral blood flow, O_2_ delivery, and finally neuronal metabolism. In the present study no significant changes were found in cardiovascular risk factors, such as awake BP, total cholesterol, and glucose levels after ET. These factors seem not to be the major determinant for changes in the CMRgl with ET in this studied group. We recently demonstrated that ET promoted a marked increase in forearm blood flow at rest and during handgrip exercise in patients with moderate to severe OSA^16^. We speculate that endothelial factors (through the release of vasodilating substances) with ET ameliorate the vascular properties and, therefore, may contribute to improve brain cerebral metabolism. In our study the improvement in CMRgl was evident in the right frontal lobe. This result is in line with the observation that cerebral brain metabolism impairment is lateralized in several sites in patients with OSA compared with healthy controls^4^. The greater injury in the right side of the brain has been reported in patients with OSA^42^. The mechanisms underlying asymmetry of metabolic changes in patients with OSA remain speculative, but the right side has a very high degree of damage to sympathetic areas and potential vascular contribution to the injury process and perhaps being more affected selected structures with extreme changes of perfusion accompanying OSA^43^. The significant reduction of sympathetic activity at resting and during mental task after ET in patients with OSA^17^ is in line with the brain metabolic changes observed in this study as evaluated by CMRgl.

The findings of this study observed in the brain metabolism may also help to explain the improvement in cognitive functioning after ET, as well as after other OSA studies using treatment with CPAP^6,20^. In the present study, ET led to a significant improvement in attentional/executive functioning, as assessed by SCWT—part 1, Trail Making Test Part B, and Frontal Assessment Battery. Executive dysfunction has been reported in patients with OSA and is responsible for decreasing cognitive abilities to control attention, inhibit inappropriate responses or behaviours (inhibit impulsivity traits), and the ability to switch between various tasks or mental sets^43^ that may affect social skills and quality of life^17^. Impaired cognitive functioning in patients with OSA has also been reported to be associated with increased risk of motor vehicle accidents and occupational hazards^44,45^. In the present study, ET promoted better performance in tests involving attention, frontal function, and executive functioning. We speculate that decrease in the CMRgl in the control group is suggestive of a progressive damage caused by intermittent hypoxia in untreated patients with OSA (Fig. 4A). In the present study changes in CMRgl in frontal area after ET correlated with changes in SCWT—part 1 in the ET group that is an attention test that is related to frontal area. The results of the present study are in line with the concept that ET has a prominent positive impact on frontal functioning in patients with moderate to severe OSA.

In line with previous studies^46,47^, ET also caused an improvement in the severity of OSA. The observation that exercise changed arousals and AHI demonstrates that ET improves sleep pattern in patients with OSA. The explanations for this exercise-induced response are uncertain. ET did not change BMI. Food intake was not limited in the present study. In this trial, participants in the control and exercise-training group were instructed to maintain the same eating habits. Thus, we believe that food intake was not a factor that influenced the results or ET compliance (85% to 100%). However, an interesting finding in our study was the effects of ET on percentage of body fat. Body fat is more accurate probably because it takes into account a person's adipose tissue rather than putting together body fat and lean muscle mass. ET can also increase lean muscle mass making it difficult to analyze through BMI alone. We can speculate that ET decreases body fat which may diminishes the upper airway collapse incidence during sleep. We found an inverse correlation between changes in maximal exercise load achieved in the maximal cardiopulmonary exercise test and changes in body fat (r = − 0.45, *P* < 0.05) in the trained group. Furthermore, ET may lead to a decrease in nocturnal rostral fluid, which also contributes to a decrease in OSA severity^14,15^. In addition, we found an inverse relationship between obstructive AHI and apnea arousal index with CMRgl after ET and an inverse relationship between changes in mean O_2_ saturation during sleep and non-rapid eye movement sleep, desaturation during arousal with changes in CMRgl. These correlations may suggest that improvement in sleep severity after ET increases CMRgl, preventing executive decline in patients with moderate to severe OSA. Thus, in addition to the several direct beneficial effects of ET on vascular and neuronal function, part of the effects of ET may be mediated by improvement in the severity of OSA.

Our study has strengths and limitations. In our study, ET and control groups were similar for age, sex, body mass index, and sedentary lifestyle, thus ruling out any potential confounding influence of these parameters on our results. In the present study all analysis were conducted using APOE ε4 allele as a covariate and the frequency of a single copy of APOE ε4 allele was similar in the control and ET groups. This control is important because recent findings from brain PET studies have suggested that CMRgl impairment could be accelerated by the presence of the APOE ε4 allele^21,48^. A single copy of the APOE ε4 allele was sufficient to determine CMRgl reductions mainly involving the frontal cortex^48,49^. The present study was planned to carry out 6 months of supervised ET performed 3 times a week, totaling 72 training sessions. Due to limitation to comply with the schedule, the exercise-trained individuals were able to participate in the training with a frequency ranging from 1 to 3 times a week. Thus, we extended the duration of the ET protocol to reach the planned target of 72 exercise sessions. Therefore, the brain changes analysis was carefully corrected for time interval between measurements. On the other hand, our ET protocol probably better reflects real life conditions. Moreover, the significant improvement in peak VO_2_ after ET indicates that the program was effective.

In summary, in sedentary patients with moderate to severe OSA, ET is associated with improvement not only in exercise capacity and OSA severity but also increased CMRgl, attention/executive functioning. Such exercise intervention may decrease the risk of developing cognitive decline in patients with OSA. ET appears to be an attractive and nonpharmacological adjuvant in the treatment of patients with moderate to severe OSA.

## Supplementary Information

Supplementary Information.

## Data Availability

The datasets generated during and/or analysed during the current study are available from the corresponding author on reasonable request.

## References

[1] Somers VK (2008). Sleep apnea and cardiovascular disease: An American Heart Association/American College of Cardiology Foundation Scientific Statement from the American Heart Association Council for High Blood Pressure Research Professional Education Committee, Council on Clinical Cardiology, Stroke Council, and Council on Cardiovascular Nursing. J. Am. Coll. Cardiol..

[2] Quan W (2018). High risk characteristics for recurrent cardiovascular events among patients with obstructive sleep apnoea in the SAVE Study. EClin. Med..

[3] Mansukhani MP, Kolla BP, Somers VK (2019). Hypertension and cognitive decline: Implications of obstructive sleep apnea. Front. Cardiovasc. Med..

[4] Yaouhi K (2009). A combined neuropsychological and brain imaging study of obstructive sleep apnea. J. Sleep Res..

[5] Daulatzai MA (2017). Cerebral hypoperfusion and glucose hypometabolism: Key pathophysiological modulators promote neurodegeneration, cognitive impairment, and Alzheimer's disease. J. Neurosci. Res..

[6] Ju G, Yoon IY, Lee SD, Kim YK, Yoon E, Kim JW (2012). Modest changes in cerebral glucose metabolism in patients with sleep apnea syndrome after continuous positive airway pressure treatment. Respiration.

[7] Kheirandish L, Gozal D, Pequignot JM, Pequignot J, Row BW (2005). Intermittent hypoxia during development induces long-term alterations in spatial working memory, monoamines, and dendritic branching in rat frontal cortex. Pediatr. Res..

[8] Gozal D, Daniel J, Dohanich GP (2001). Behavioral and anatomical correlates of chronic episodic hypoxia during sleep in the rat. J. Neurosci..

[9] Sforza E, Roche F (2012). Sleep apnea syndrome and cognition. Front. Neurol..

[10] O'Hara R (2005). Nocturnal sleep apnea/hypopnea is associated with lower memory performance in APOE epsilon4 carriers. Neurology.

[11] Yaffe K (2011). Sleep-disordered breathing, hypoxia, and risk of mild cognitive impairment and dementia in older women. JAMA.

[12] Devita M (2019). Associations between the apnea-hypopnea index during REM and NREM sleep and cognitive functioning in a cohort of middle-aged adults. J. Clin. Sleep Med..

[13] Cosentino FI (2008). The APOE epsilon4 allele increases the risk of impaired spatial working memory in obstructive sleep apnea. Sleep Med..

[14] Iftikhar IH, Kline CE, Youngstedt SD (2014). Effects of exercise training on sleep apnea: A meta-analysis. Lung.

[15] Andrade FM, Pedrosa RP (2016). The role of physical exercise in obstructive sleep apnea. J. Bras. Pneumol..

[16] Guerra RS (2019). Exercise training increases metaboreflex control in patients with obstructive sleep apnea. Med. Sci. Sports Exerc..

[17] Goya TT (2016). Increased muscle sympathetic nerve activity and impaired executive performance capacity in obstructive sleep apnea. Sleep.

[18] Ferreira-Silva R (2018). Vascular response during mental stress in sedentary and physically active patients with obstructive sleep apnea. J. Clin. Sleep Med..

[19] Araújo CEL (2021). Effects of exercise training on autonomic modulation and mood symptoms in patients with obstructive sleep apnea. Braz. J. Med. Biol. Res..

[20] Canessa N, Castronovo V, Cappa SF (2011). Obstructive sleep apnea: Brain structural changes and neurocognitive function before and after treatment. Am. J. Respir. Crit. Care Med..

[21] Kisler K, Nelson AR, Montagne A, Zlokovic BV (2017). Cerebral blood flow regulation and neurovascular dysfunction in Alzheimer disease. Nat. Rever. Neurosci..

[22] Fan J (2019). The contribution of genetic factors to cognitive impairment and dementia: Apolipoprotein E gene, gene interactions, and polygenic risk. Int. J. Mol. Sci..

[23] Luo Y, Tan L, Therriault J, Zhang H, Gao Y, The Alzheimer’s Disease Neuroimaging Initiative (2021). The role of apolipoprotein E epsilon 4 in early and late mild cognitive impairment. Eur. Neurol..

[24] Wang HK (2006). Apolipoprotein E, angiotensin-converting enzyme and kallikrein gene polymorphisms and the risk of Alzheimer's disease and vascular dementia. J. Neural. Transm..

[25] Ueno LM (2009). Effects of exercise training in patients with chronic heart failure and sleep apnea. Sleep.

[26] Drager LF, Ueno LM, Lessa PS, Negrão CE, Lorenzi-Filho G, Krieger EM (2009). Sleep-related changes in hemodynamic and autonomic regulation in human hypertension. J. Hypertens..

[27] Iber C, Ancoli-Israel S, Chesson A, Quan SF (2007). The AASM Manual for the Scoring of Sleep and Associated Events: Rules, Terminology and Technical Specifications.

[28] Folstein MF, Folstein SE, McHugh PR (1975). “Mini Mental State” A practical method for grading the cognitive state of patients for the clinician. J. Psychiatry Res..

[29] Malloy-Diniz LF, Lasmar VA, Gazinelli LD, Fuentes D, Salgado JV (2007). The Rey Auditory-Verbal Learning Test: Applicability for the Brazilian elderly population. Rev. Bras. Psiquiatr..

[30] Beato RG, Nitrini R, Formigoni AP, Caramelli P (2007). Brazilian version of the Frontal Assessment Battery (FAB): Preliminary data on administration to healthy elderly. Dement. Neuropsychol..

[31] Campanholo KR (2014). Performance of an adult Brazilian sample on the Trail Making Test and Stroop Test. Dement. Neuropsychol..

[32] Zimmermann N, Cardoso CO, Trentini CM, Grassi-Oliveira R, Fonseca RP (2015). Brazilian preliminary norms and investigation of age and education effects on the Modified Wisconsin Card Sorting Test, Stroop Color and Word test and Digit Span test in adults. Dement. Neuropsychol..

[33] Golden C, Freshwater S (2002). A Manual for the Adult Stroop Color and Word Test.

[34] Wechsler D (1999). Wechsler Abbreviated Scale of Intelligence (WASI).

[35] Gonçalves DM, Stein AT, Kapczinski F (2008). Avaliação de desempenho do Self-Reporting Questionnaire como instrumento de rastreamento psiquiátrico: Um estudo comparativo com o Structured Clinical Interview for DSM-IV-TR. Cad. Saúde Pública..

[36] Beck AT, Epstein N, Brown G, Steer RA (1988). An inventory for measuring clinical anxiety: Psychometric properties. J. Consult. Clin. Psychol..

[37] Beck AT, Ward CH, Mendelson M, Mock J, Erbaugh J (1961). An inventory for measuring depression. Arch. Gen. Psychiatry.

[38] Dean, A.G., Sullivan, K.M. & Soe, M.M. *OpenEpi**: **Open Source Epidemiologic Statistics for Public Health*. Versão 3.03a ed. (2015).

[39] Kline CE (2011). The effect of exercise training on obstructive sleep apnea and sleep quality: A randomized controlled trial. Sleep.

[40] Kline CE (2012). Exercise training improves selected aspects of daytime functioning in adults with obstructive sleep apnea. J. Clin. Sleep Med..

[41] Beebe DW, Gozal D (2002). Obstructive sleep apnea and the prefrontal cortex: Towards a comprehensive model linking nocturnal upper airway obstruction to daytime cognitive and behavioral deficits. J. Sleep Res..

[42] Harper RM, Kumar R, Macey PM, Woo MA, Ogren JA (2014). Affective brain areas and sleep-disordered breathing. Prog. Brain Res..

[43] Logue SF, Gould TJ (2014). The neural and genetic basis of executive function: Attention, cognitive flexibility, and response inhibition. Pharmacol. Biochem. Behav..

[44] Karimi M (2015). Attention deficits detected in cognitive tests differentiate between sleep apnea patients with or without a motor vehicle accident. Sleep Med..

[45] Hong S (2014). A case of obstructive sleep apnea and assessments of fitness for work. Ann. Occup. Environ. Med..

[46] Maki-Nunes C (2015). Diet and exercise improve chemoreflex sensitivity in patients with metabolic syndrome and obstructive sleep apnea. Obesity.

[47] Bughin F (2020). Effects of an individualized exercise training program on severity markers of obstructive sleep apnea syndrome: A randomized controlled trial. Sleep Med..

[48] Mosconi L (2004). Age and ApoE genotype interaction in Alzheimer's disease: An FDG-PET study. Psychiatry Res..

[49] Mosconi L (2003). Brain metabolic differences between sporadic and familial Alzheimer’s disease. Neurology.

