# Supplementary material for: Effects of exercise training on brain metabolism and cognitive functioning in sleep apnea
Source: Sci Rep. 2022 Jun 8;12:9453. doi: 10.1038/s41598-022-13115-2 (PMC9177702; doi:10.1038/s41598-022-13115-2)
Supplement: Supplementary file 1 — Supplementary Information. [file 41598_2022_13115_MOESM1_ESM.pdf]

# Effects of exercise training on brain metabolism and cognitive functioning in sleep apnea

***Short title:*** *Exercise training on brain health*

Linda M. Ueno-Pardi, Fabio L. Souza-Duran, Larissa Matheus, Amanda G. Rodrigues, Eline R.F. Barbosa, Paulo J. Cunha, Camila G. Carneiro, Naomi A. Costa, Carla R. Ono

Carlos A. Buchpiguel, Carlos E. Negrão, Geraldo Lorenzi-Filho, Geraldo Busatto-Filho

## Supplemental Digital Content 1

## Cognitive Evaluation

The Mini-Mental State Examination, which is a screening neurocognitive tool that covers domains, such as orientation, memory, registration, recall, constructional ability, language, and the ability to understand and follow commands, was administered. The Rey Auditory Verbal Learning Test (RAVLT) consists of 15 nonrelated words that should be orally repeated by the patients in 5 consecutive trials (immediate verbal memory and learning abilities), and remembered after 30 minutes (delayed recall) (S1). The Frontal Assessment Battery has 6 subtests that assess the conceptualization (abstraction), lexical fluency (mental flexibility), motor programming, sensitivity to interference (tendency to distraction), inhibitory control, and autonomy. Each of the subtests is equivalent to a maximum of 3 points. Together, the 6 subtests will total 18 points, which is the maximum possible score obtained in the Frontal Assessment Battery. The Frontal Assessment Battery has been proposed recently as a brief diagnostic tool to be used in cases of dysexecutive syndrome (S2). The Trail Making Test – Part A consists of a series of numbers that have to be linked with a pencil by the patient following the increasing order of the numbers. In the Trail Making Test – Part B, the participant alternates between numbers and letters, with numbers in a crescent order and letters in alphabetical order. The Forward Digits test requires the verbal repetition of digits in the same order, whereas in the Backward digits test the participant is asked to repeat the sequence of numbers in the inverse order. In the Digit Symbol test, a visual key consisting of 110 paired geometric figures and numbers is provided. Participants are asked to apply a key to supply the proper number that is associated with the specific symbol. The outcome is the number of correct responses in 90 seconds. The Stroop Color Word Test (SCWT) (S3) is made up of 3 cards containing 6 lines with 4 items: the first (SCWT – Part 1) made up of colored cards (green, pink, blue, and

brown); the second (SCWT – Part 2) consisting of neutral words written with the colors of the tags, and the third (SCWT – Part 3) with the names of colors written in conflicting colors with the print. The participant is asked to verbalize the printed colors of each card as soon as possible. The time is started right after the instructions and the time it takes the subject to read each card is recorded. In this study, shifting relates to the ability to shift between operations, tasks, or mental sets in order to perform goal-related behavior, whereas inhibition refers to the ability to inhibit responses or behaviors if a reaction is inappropriate in a given context. The updating function is used to actively manipulate information (clearing and updating working memory) and to monitor cognitive processing in real-time (S4, S5). Some models consider shifting, inhibition, and updating function as a main domain of executive function (S4)

## Neuroimaging

### *$^{18}$FDG-PET and magnetic resonance (MR) data acquisition and analysis*

The MR imaging data were acquired both at baseline and after follow-up using a 1.5-T MR imaging scanner (SIEMENS MAGNETOM Espree syngo MR B15, Germany). The protocol included acquisition of contiguous sagittal images across the entire brain using a T1-3D MPRAGE sequence with the following parameters: TE = 3.62 ms, TR = 2400 ms, flip angle = 8, acquisition matrix = 192x192 pixels, and a voxel size of 1.25 × 1.25 × 1.2 mm (160 slices). All images were visually checked by an experienced radiologist with the purpose of identifying artifacts and the presence of silent gross brain lesions, such as tumors and silent infarcts (stroke or lacunar infarcts). PET data were acquired within 1 week after MR imaging acquisition by using a dedicated lutetium oxyorthosilicate-16-section FDG-

PET scanner (Biograph-16; Siemens, Erlangen, Germany) with a spatial resolution of 2.5-mm FWHM, 3.38-mm section thickness, and 500-mm axial FOV. Data were collected in 256 × 256 matrices, by using a smoothing factor of 5. Voxel size was set to 1.06 × 1.06 × 3.38 mm (x, y, z). Interactive reconstruction was applied by using 6.0 subsets and an interaction number of 16. Images were corrected for attenuation by using the computed tomography algorithm.

**Neuroimage Processing**

First, the datasets of 18FDG-PET and T1-MR were converted from DICOM to NIfTI format using DCM2NII software (http://www.cabiatl.com/mricro/mricron/dcm2nii.html). 18FDG-PET images were co-registered to the T1-MR datasets of the same individual using the PMOD software tool (version 3.4, PMOD Technologies Ltd., Zurich, Switzerland). Briefly, the individual MR image was oriented so that the anterior commissure was aligned at the origin of the three-dimensional Montreal Neurological Institute (MNI) coordinate system. The transformation parameters of the MR were applied to the corresponding $^{18}$FDG-PET images. All images were manually oriented and visually checked for coregistration. Coregistered $^{18}$FDG-PET images were corrected for partial volume effects (PVEs) through the modified Meltzer method, using PVELAB software (http://pveout.ibb.cnr.it/PVEOut_Software.htm), described in detail by Quarantelli et al. (S6).

All the T1-MRI and $^{18}$FDG-PET images were processed using Statistical Parametric Mapping, version 12 (SPM12; https://www.fil.ion.ucl.ac.uk/spm/), implemented in MATLAB software (The Math-Works, Natick, MA, USA). First, the CAT12 toolbox (http://www.neuro.uni-jena.de/cat/) was used to process pre- and post-intervention T1-MRI

data. The CAT12 for SPM offers an alternative approach that allows more accurate analysis of brain regions (S7). Then, the T1-MR datasets of each individual were spatially normalized to the standard MNI space using the Diffeomorphic Anatomical Registration Through Exponentiated Lie Algebra (DARTEL) tool to create a set of group-specific templates. Subsequently, the steps used in the spatial normalization of T1-MR datasets of each individual were applied to the PVE-corrected $^{18}$FDG-PET images to achieve spatial normalization to MNI space. Thus, the spatially normalized, PVE-corrected $^{18}$FDG-PET images were smoothed with an 8-mm full-width-at-half-maximum (FWHM) Gaussian filter.

**Statistical analysis**

Default parameters were used, and quality checks were conducted. The CMRgl peak values for each subject (voxel values extracted from the coordinate of maximal statistical significance in the voxel-wise group comparisons) were analyzed. Findings of the hypothesis driven analyses, voxel-wise inspections of each SPM were conducted in constrained a priori brain regions using a small volume correction (SVC) tool. Such composite anatomical masks were derived from spatially normalized region-of-interest (ROI) masks available within the Anatomical Automatic Labeling (AAL) SPM toolbox. ROI masks were used for each hemisphere, involving the frontal lobe (anterior, middle, posterior, superior middle), opercular, and triangular inferior. The SVC analyses were reported as significant if surviving family-wise error correction for multiple comparisons ($P_{FWE\text{-}corr} \leq 0.05$) over the respective brain region. Only clusters with a minimum of 20 voxels were reported.

**References**

S1) Malloy-Diniz, L.F., Lasmar, V.A.P., Gazinelli, L. de S.R., Fuentes, D. & Salgado, J.V. The Rey Auditory-Verbal Learning Test: applicability for the Brazilian elderly population. *Rev. Bras. Psiquiatr.* **29**, 324–329 (2007).

S2) Dubois, B., Slachevsky, A., Litvan, I., & Pillon, B. The FAB: A Frontal Assessment Battery at bedside. *Neurology.* **55**(11), 1621–1626 (2000).

S3) Campanholo, K.R.  et al. Performance of an adult Brazilian sample on the Trail Making Test and Stroop Test. *Dement. Neuropsychol.* **8**(1), 26–31 (2014).

S4) Miyake, A., Friedman, N. P., Emerson, M. J., Witzki, A. H., Howerter, A., & Wager, T. D. The unity and diversity of executive functions and their contributions to complex “frontal lobe” tasks: A latent variable analysis. *Cogn. Psychol*. **41**(1), 49–100 (2000).

S5) Mas, M., Chambaron, S., Chabanet, C., & Brindisi, M.C. Inhibition and shifting across the weight status spectrum. *Appl. Neuropsychol. Adult*. **21**, 1–8 (2022).

S6) Quarantelli, M. *et al*. Integrated software for the analysis of brain PET/SPECT studies with partial-volume-effect correction. *J. Nucl. Med.* **45**, 192–201 (2004).

S7) Farokhian, F., Beheshti, I., Sone, D. & Matsuda, H. Comparing CAT12 and VBM8 for detecting brain morphological abnormalities in temporal lobe epilepsy. *Front. Neurol.* **24(**8), 428 (2017).
